# Measured thoracic gas volume using the bod pod air displacement plethysmography system versus three predictions

**DOI:** 10.1007/s00421-025-05863-6

**Published:** 2025-06-25

**Authors:** Dale R. Wagner, Edward M. Heath, Steven Spencer, Alyssa Evans, Jacob McBride, Tate Burch

**Affiliations:** 1 Kinesiology & Health Science Department, Utah State University, 7000 Old Main Hill, Logan, UT 84322-7000 USA; 2 Nutrition, Dietetics & Food Sciences, Utah State University, Logan, UT USA

**Keywords:** Athlete, Body composition, Body volume, Lung volume, Validity

## Abstract

**Purpose:**

Bod Pod air displacement plethysmography is a common method to estimate body fat percentage (%BF). To determine body volume from this method, thoracic gas volume (TGV) must be quantified. Measured TGV was compared to three predictions: the default prediction formula in the Bod Pod software from Crapo (CRAPO), a relatively new prediction that relies on height and body mass as the predictors from Ducharme (DUCHARME), and a child-specific formula from Fields (FIELDS).

**Methods:**

University club sport athletes (121 men, 80 women) completed the breathing maneuver in the Bod Pod necessary to obtain measured TGV. Values from the three TGV predictions were compared to measured TGV with a one-way repeated measures ANOVA. Linear regression was used to obtain standard error of estimate (*SEE*), and Bland–Altman plots were used to evaluate bias.

**Results:**

Significant (*P* < .001) bias exists for all three prediction equations such that individuals with small TGV are overpredicted and those with large TGV are underpredicted. *SEE*s were similar (0.63–0.66 L) for all three equations. On average, CRAPO and FIELDS underestimated (*P* < .001) measured TGV by -0.25 L and -0.43 L, respectively, but the difference between DUCHARME and measured TGV (0.07 L) was not significant (*P* = .813). There were fewer large errors (≥ 2%BF) from DUCHARME (25%) than from CRAPO (31%) or FIELDS (37%).

**Conclusion:**

TGV should be measured. If it must be predicted, DUCHARME offers a slightly better prediction than CRAPO or FIELDS for young adult athletes, particularly those at the extremes of the TGV continuum.

## Introduction

The Bod Pod air displacement plethysmography (ADP) system was introduced in 1995 with a technical paper detailing the science supporting the ADP method for the measurement of body volume (Dempster and Aitkens 1995) and a validity paper comparing the Bod Pod to hydrostatic weighing (McCrory et al. 1995). Given the ease of measurement for the Bod Pod relative to hydrostatic weighing (e.g., participant does not get wet, no maximal exhalation under water, faster procedure, no tank maintenance) it is not surprising that the ADP method has largely supplanted underwater weighing as the laboratory method for determining body volume. For example, combining the search term “body composition” with “air displacement plethysmography” versus “hydrostatic weighing” or “hydrodensitometry” using the Scopus database results in nearly five times the number of manuscripts for ADP compared to hydrodensitometry over the past 20 years.

Accuracy of the body volume measurement from ADP requires accounting for the participant’s skin surface and lung volume (Dempster and Aitkens 1995; McCrory et al. 1995). The Bod Pod automatically accounts for skin surface by calculating body surface area from the participant’s height and weight using the Dubois formula (Dubois and Dubois 1916) then converts this to a surface area artifact value (Dempster and Aitkens 1995). Thoracic gas volume (TGV) is measured with a breathing maneuver in which the participant gently puffs against an occluded airway at mid-exhalation. Dempster and Aitkens (1995) detail how the Bod Pod derives TGV from this breathing maneuver. Consequently, the raw body volume (Vb) is corrected for surface area artifact and TGV (Dempster and Aitkens 1995):

$${\text{Vb}} = {\text{Vb}}_{{{\text{raw}}}} - {\text{surface}}\; {\text{area}}\; {\text{artifact}} + 40\% {\text{TGV}}.$$

Measuring TGV requires a single-use filter/breathing tube kit (i.e., small additional expense) and adds time to the Bod Pod procedure. Further, the breathing maneuver can be difficult for some participants to master. In fact, some people cannot achieve an acceptable result even after multiple trials. Thus, there is an option in the Bod Pod software to predict TGV using the formula of Crapo et al. (1982) (CRAPO).

McCrory et al. (1998) reported no significant difference between measured TGV and CRAPO nor did the predicted TGV significantly change the estimate of body fat percentage (%BF). Others have reported similar findings of no mean difference between measured TGV and CRAPO (Collins and McCarthy 2003; Otterstetter et al. 2015; Wagner 2015). However, when considering individual error rather than group means, the predictive accuracy of CRAPO is questionable; specifically, there is a systematic overestimation of TGV for individuals with a small TGV and an underestimation of TGV for individuals with a large TGV (Ducharme et al. 2021; Minderico et al. 2008; Wagner 2015). Given this systematic bias, Ducharme et al. (2022) developed and validated a new TGV prediction (DUCHARME) on young adults. A child-specific TGV prediction created by Fields et al. (2004) (FIELDS) from a sample of 6- to 17-year-old children also exists. The purpose of the present study was to evaluate the predictive accuracy of all three TGV prediction formulas (CRAPO, DUCHARME, and FIELDS) against measured TGV in a sample of young adult athletes. We hypothesized that the new DUCHARME formula would be valid and superior to the other two prediction equations for estimating the TGV of our sample.

## Methods

### Participants

This investigation was part of a multicomponent body composition study of university club sport athletes (Wagner et al. 2025). The participants in the present study included all of the participants in the multicomponent study (Wagner et al. 2025) who had a measured TGV. All participants were ≥ 18 y of age. Men were recruited from 16 club sports (baseball, climbing, cycling, gymnastics, ice hockey, jump rope, lacrosse, pickleball, powerlifting, racquetball, rodeo, rugby, soccer, swimming, ultimate, and volleyball). Women were recruited from 13 club sports (climbing, cycling, figure skating, gymnastics, jump rope, lacrosse, powerlifting, rodeo, rugby, soccer, swimming, ultimate, and volleyball). The study was approved by Utah State University’s Institutional Review Board (protocol #12253), and all participants gave written informed consent prior to their participation.

### Procedures

Height was measured to the nearest 0.1 cm with a wall-mounted stadiometer (Seca 216, Seca Corp., Ontario, CA). Body mass was measured to the nearest 0.01 kg with the Bod Pod scale (Cosmed USA, Inc., Concord, CA) as part of the Bod Pod testing procedure. Consistent with the manufacturer’s guidelines, participants wore compression clothing including a swim cap.

The Bod Pod ADP system (version 5.4.5, Cosmed USA, Inc., Concord, CA) was calibrated with the manufacturer-provided calibration cylinder, and body volume was measured according to the manufacturer’s instructions. This included sitting in the Bod Pod for consecutive trials of 20 s each to achieve two body volume measurements within 150 mL precision. An additional measurement followed with participants breathing through a tube while wearing a nose clip to obtain measured TGV. When directed by a prompt in the Bod Pod software, the participant performed a panting or puffing maneuver. This “puffing” maneuver consisted of three gentle puffs through the breathing tube as if fogging a pair of glasses. The puffing coincided with mid-exhalation and momentary occlusion of the airway. The Bod Pod software provided a “merit score” to indicate if the breathing maneuver was successfully performed. If successful, this value was recorded as measured TGV and concluded the data collection; if not successful, the breathing sequence was repeated. Participants who could not achieve a valid measured TGV after 5 trials were removed from analysis. Body density was automatically calculated from the Bod Pod software as body mass divided by body volume; subsequently, body density was converted to %BF using the Siri (1961) formula.

Bod Pod software calculations to determine measured TGV are provided elsewhere (McCrory et al. 1998). The published TGV prediction equations of Crapo et al. (1982), Ducharme et al. (2022), and Fields et al. (2004) (Table 1) were calculated. Using the “modify subject results” feature of the Bod Pod software, the %BF estimation would automatically change as the TGV value was changed from measured TGV to each of the three predicted TGV values.

**Table 1:** Thoracic gas volume (TGV) prediction equations

References	Equation
Crapo et al. (1982)	Men: TGV = FRC + 0.5 (TV); where FRC = 0.0472 (ht) + 0.0090 (age) – 5.290 Women: TGV = FRC + 0.5 (TV); where FRC = 0.0360 (ht) + 0.0031 (age) – 3.182
Ducharme et al. (2022)	TGV = 0.615338 (sex; 0 = women, 1 = men) + 0.056267 (ht) – 0.011006 (wt) – 5.358839
Fields et al. (2004)	Boys: TGV = 0.00056 (ht^2^) – 0.12422 (ht) + 8.15194 Girls: TGV = 0.00044 (ht^2^) – 0.09220 (ht) + 6.00305

*TGV* thoracic gas volume in L, *FRC* functional residual capacity in L, *TV* tidal volume in L (assumed to be 1.2 L for men and 0.7 L for women) *ht* height in cm, *wt* weight in kg

### Statistical analyses

Data were analyzed for the entire sample as well as for the sexes separately. The values from the three TGV prediction equations were compared to measured TGV with a one-way repeated-measures analysis of variance (ANOVA), and a Bonferroni post-hoc test followed a significant omnibus F. Statistical significance was accepted as *P* < 0.05. Additionally, linear regression was used to determine the standard error of estimate (*SEE*) of each prediction equation. Individual error and bias were evaluated with Bland and Altman (1999) plots. Analysis of error frequencies was done to determine the percentage of participants for whom the prediction error was equal to or greater than ± 2% BF. All analyses were done using SPSS (version 29, IBM, Inc., Armonk, NY).

## Results

From a sample of 231 athletes, 201 (87.0%) successfully completed the puffing maneuver for measured TGV. All TGV analyses were done on these 201 athletes (121 men, 80 women). Descriptive characteristics of the sample are in Table 2. Additional descriptive details of the multicomponent model study from which this TGV dataset is derived, such as body composition data by sport and multicomponent model statistics, are available elsewhere (Wagner et al. 2025).

**Table 2:** Descriptive characteristics of the sample (*N* = 201). Data are mean ± SD with minimum to maximum values in parentheses

	Age (y)	Height (cm)	Mass (kg)	BMI (kg/m^2^)
Men (n = 121)	21.4 ± 1.9 (18–25)	179.1 ± 6.9 (162.1–194.6)	79.6 ± 12.9 (57.3–117.1)	24.8 ± 3.5 (16.9–36.4)
Women (n = 80)	20.4 ± 1.9 (18–28)	166.8 ± 7.2 (149.2–180.5)	66.6 ± 11.9 (46.2–106.9)	23.9 ± 3.5 (18.1–34.8)
Total (*N* = 201)	21.0 ± 2.0 (18–28)	174.2 ± 9.3 (149.2–194.6)	74.4 ± 14.0 (46.2–117.1)	24.4 ± 3.5 (16.9–36.4)

*BMI* body mass index

**Fig. 1:**
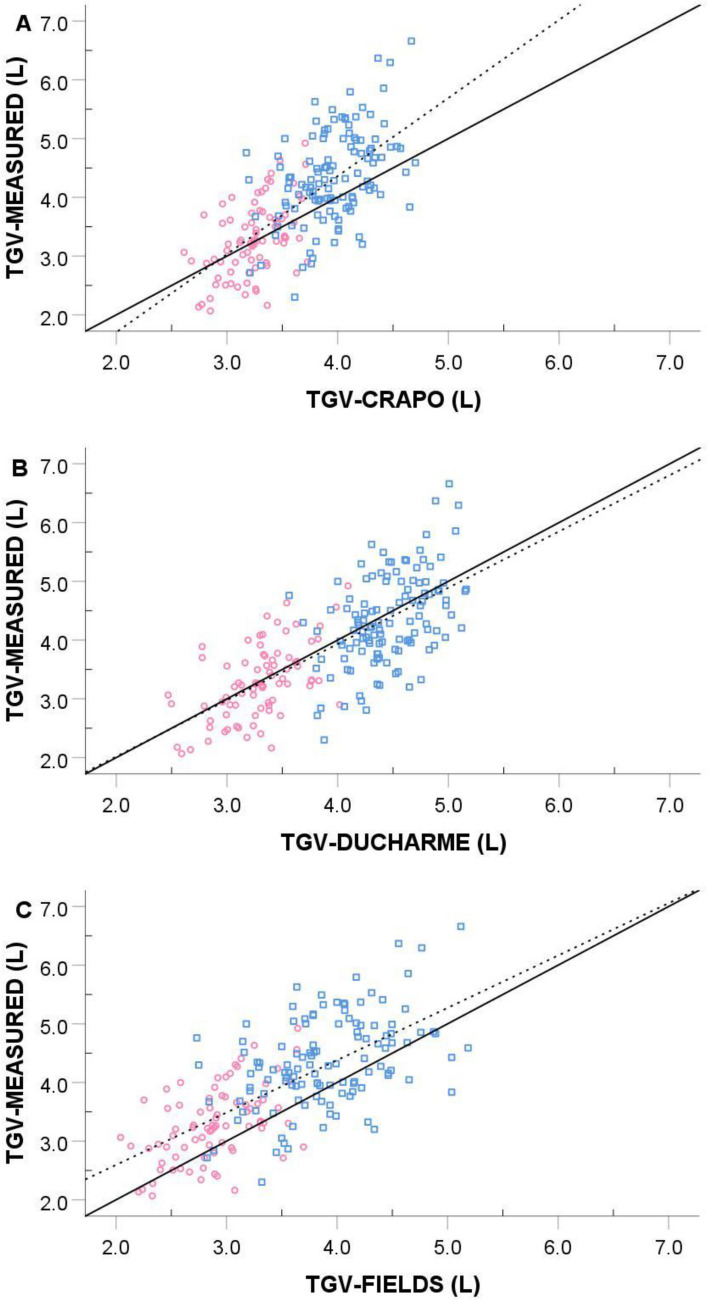
Relationship between measured thoracic gas volume and predicted values from **A** CRAPO, **B** DUCHARME, and **C** FIELDS. Solid line is the line of identity, and the dashed line is the regression line. Men are represented as blue squares, and women are represented as pink circles

**Fig. 2:**
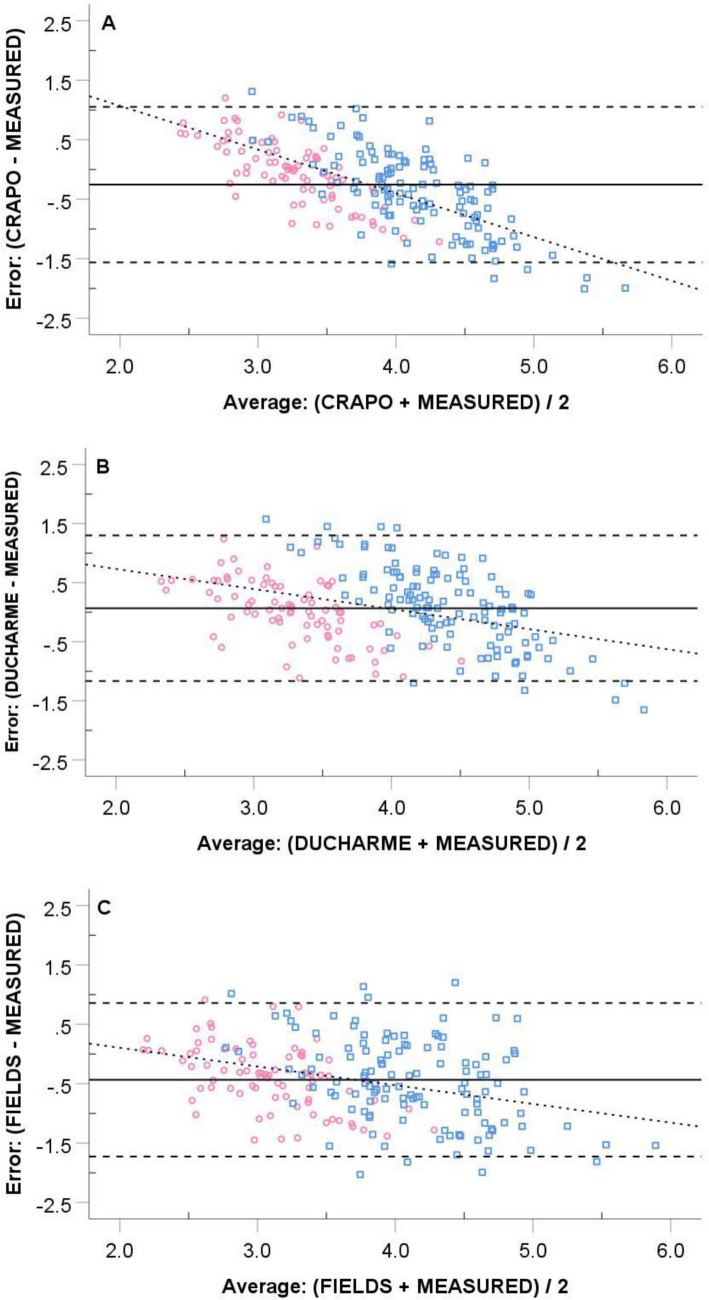
Bland and Altman plots of the error (TGV predicted minus measured TGV) against the average for the thoracic gas volume predictions of **A** CRAPO, **B** DUCHARME, and **C** FIELDS. Solid line is the constant error. Horizontal dashed lines are the 95% limits of agreement. The dashed line fit to the data represent the systematic error. Men are represented as blue squares, and women are represented as pink circles

Comparisons of the TGV and %BF values from the different prediction equations are in Table 3. For the total sample (N = 201), the one-way repeated-measures ANOVA was significant (*P* < 0.001) for TGV, as CRAPO and FIELDS underestimated measured TGV by -0.25 L and -0.43 L, respectively; however, the difference between DUCHARME and measured TGV (0.07 L) was not significant (*P* = 0.813). Similarly, %BF was significantly (*P* < 0.001) underestimated when CRAPO (-0.7% BF) and FIELDS (-1.3% BF) predictions were used, but the difference in %BF was not significant between measured TGV and DUCHARME (0.2% BF, *P* = 1.000). The *SEE*s for both TGV and %BF were nearly identical across the three predictions. Despite similar *SEE*s, the regression line and line of identity matched nicely for DUCHARME, but this was not the case for CRAPO and FIELDS (Fig. 1). The relationship between the error and average of the measured and predicted TGV was significant (*P* < 0.001) and negative for all three prediction formulas (Fig. 2): CRAPO (*r* = −0.697), DUCHARME (*r* = −0.392), and FIELDS (*r* = −0.348). This indicates a bias such that individuals with small measured TGV are overestimated by the prediction formulas, and individuals with large measured TGV are underestimated by the prediction formulas. About a quarter of participants had prediction errors ≥ 2% BF when the DUCHARME formula was used, and this value exceeded 30% for both CRAPO and FIELDS.

**Table 3:** Comparison of measured thoracic gas volume to the predictions

TGV formula	TGV (L)	*SEE* (L)	%BF	*SEE* (%BF)	Errors ≥ ± 2%BF
Men (n = 121)					
Measured	4.36 ± 0.78	–	14.2 ± 6.6	–	–
Crapo et al. (1982)	3.96 ± 0.33*	0.71	13.1 ± 7.1*	1.6	33.1%
Ducharme et al. (2022)	4.46 ± 0.35	0.69	14.4 ± 6.8	1.7	24.8%
Fields et al. (2004)	3.89 ± 0.53*	0.70	12.9 ± 7.1*	1.7	38.0%
Women (n = 80)					
Measured	3.28 ± 0.63	–	24.8 ± 7.0	–	–
Crapo et al. (1982)	3.24 ± 0.26	0.55	24.6 ± 7.6	1.5	28.8%
Ducharme et al. (2022)	3.29 ± 0.35	0.53	24.8 ± 7.4	1.5	25.0%
Fields et al. (2004)	2.89 ± 0.39*	0.55	23.5 ± 7.8*	1.5	36.3%
Total (N = 201)					
Measured	3.93 ± 0.90	–	18.4 ± 8.5	–	–
Crapo et al. (1982)	3.67 ± 0.47*	0.65	17.7 ± 9.2*	1.6	31.3%
Ducharme et al. (2022)	3.99 ± 0.67	0.63	18.6 ± 8.7	1.7	24.9%
Fields et al. (2004)	3.49 ± 0.69*	0.66	17.1 ± 9.0*	1.7	37.3%

*SEE* standard error of estimate

* p < 0.001 compared to measured value

The results for the men paralleled those for the total sample. CRAPO and FIELDS significantly (*P* < 0.001) underestimated the measured TGV of men, but DUCHARME was not significantly different (*P* = 0.640) from the measured value. Consequently, the %BF of men was significantly (*P* < 0.001) underestimated by an average of 1.0% BF and 1.3% BF when using the CRAPO and FIELDS predictions, respectively. On average, DUCHARME resulted in a %BF estimation that did not differ from measured TGV (*P* = 0.600). For the women, the child-specific equation of Fields et al. (2004) significantly (*P* < 0.001) underestimated measured TGV by -0.39 L, leading to an underestimation of %BF by 1.3%. However, on average, there was no difference between measured TGV and CRAPO (*P* = 0.512) or DUCHARME (*P* = 0.825). Given the smaller lung volumes for women compared to men, the *SEE*s for TGV were slightly smaller for women than men. However, all of the prediction formulas produced similar *SEE*s. The pattern of the prediction formulas overestimating measured TGV for individuals with small TGVs and underestimating those with large TGVs persisted when men and women were analyzed separately.

## Discussion

The results partially support our hypothesis that the new DUCHARME formula offers a better estimation of measured TGV for young adults than the prediction formula in the Bod Pod software (CRAPO) or the child-specific formula of Fields et al. (2004) (FIELDS). DUCHARME was the only formula that produced a group mean that was not significantly different from measured TGV. The regression line closely matched the line of identity. Additionally, DUCHARME resulted in fewer errors exceeding ± 2% BF compared to the other prediction formulas. However, the *SEE*s were similar across all three prediction equations. Further, a bias exists for all three equations such that TGV is overestimated in people with a small TGV and underestimated in people with a large TGV. The magnitude of error is somewhat blunted with DUCHARME compared to CRAPO, but the bias is still present. Thus, prediction errors can be large for people with small or large TGVs.

The prediction formula in the Bod Pod software is from Crapo et al. (1982) (CRAPO). Numerous investigators reported no significant mean difference between CRAPO and measured TGV (Collins and McCarthy 2003; McCrory et al. 1998; Otterstetter et al. 2015; Wagner 2015). However, consistent with the findings in the present study, other investigators reported lower values for CRAPO compared to measured TGV, leading to an underestimation of %BF when this prediction formula is used (Ducharme et al. 2021; Miller et al. 2023). Ducharme et al. (2021) noted a sex-specific difference might exist for CRAPO such that this formula underestimates men but is valid for women. Correspondingly, we found the mean difference between CRAPO and measured TGV for the women in the present study was not significant, and the underestimation was more pronounced in men. Close inspection of the studies that reported no significant mean difference between CRAPO and measured TGV (Collins and McCarthy 2003; McCrory et al. 1998; Otterstetter et al. 2015; Wagner 2015) revealed that all of them had a preponderance of women; whereas, the present investigation and those that found CRAPO underestimates measured TGV included samples that were either exclusively (Miller et al. 2023) or predominantly (Ducharme et al. 2021) men.

More important than the mean difference is the issue of bias in the CRAPO prediction. Consistent with the present investigation, multiple researchers have reported an increasing magnitude of error as CRAPO gets further away from a typical value (Ducharme et al. 2021; Minderico et al. 2008; Wagner 2015). Ducharme et al. (2021) noted that women with a measured TGV ≥ 3.5 L and men with a measured TGV ≥ 4.5 L would be underpredicted when using the CRAPO formula. We concur. Further, women with measured TGV ≤ 2.7 L and men with measured TGV ≤ 3.3 L are likely to be overpredicted by > 0.5 L when using the CRAPO formula.

The over- and underestimation bias evident in CRAPO (see Fig. 2a) led Ducharme et al. (2022) to create a new TGV prediction formula based on height, body mass, and sex rather than assumptions about tidal volume and functional residual capacity used by Crapo et al. (1982). During the validation of their equation, Ducharme et al. (2022) reported that a bias still existed for their formula, but the prediction errors were less than those of CRAPO. The findings from the present study are consistent with those of Ducharme et al. (2022); a bias still exists for DUCHARME, but the constant error and percentage of participants with %BF errors ≥ 2% BF is less when using the DUCHARME formula compared to CRAPO.

Regarding FIELDS, we were curious as to how a formula developed from a sample of 6- to 17-year old children would perform when applied to a sample of young adults. Not surprisingly, measured TGV was underpredicted by nearly 0.5 L, leading to an underestimation of %BF. However, the S*EE*s from the regression analysis and bias from the Bland and Altman (1999) plots were similar between FIELDS and DUCHARME; thus, an adjustment to the y-intercept (see Fig. 1c) or constant error might improve the suitability of this child-specific formula for adults. Fields et al. (2004) developed FIELDS because CRAPO overestimated measured TGV in their sample of children. Other investigators have also found FIELDS to be more suitable than CRAPO when predicting the TGV of children (Higgins et al. 2006; Holmes et al. 2011; Radley et al. 2007).

Both Fields et al. (2004) and Ducharme et al. (2022) highlighted a strong relationship between height and measured TGV in the derivation of their formulas. Consequently, height is the primary predictor variable in each of their TGV prediction formulas (see Table 1). Fields et al. (2004) reported Pearson correlations of 0.79 and 0.89 for the girls and boys, respectively, in their study of children. Similarly, Ducharme et al. (2022) reported a height:TGV relationship of 0.76 in their validation sample of 140 young adults. In the present study, the height:TGV relationship was not as strong at 0.67. This weaker relationship between height and measured TGV in the present study might account for the slightly larger prediction errors that we observed compared to what was reported by Ducharme et al. (2022). Ducharme et al. (2022) reported an *SEE* of 0.56 L for their prediction equation, whereas the *SEE* for DUCHARME in our study was 0.63 L.

As noted at the beginning of the results section, even after five trials and extensive coaching 13% of the recruited participants were unable to successfully perform the puffing maneuver required for measured TGV. Few researchers report the failure rate to successfully complete the measured TGV procedure. Of those that have acknowledged it, the failure rate in adults has ranged from 9 to 26% (Anderson 2007; Miller et al. 2023; Wagner 2015) with a failure rate of 31% reported in children (Lockner et al. 2000). The inability of some individuals to produce an acceptable “merit” score for TGV according to the Bod Pod software necessitates the use of a predicted value. This further highlights the importance of this research and the need for accurate TGV prediction formulas.

There are several strengths to this study. First, it is the only known study to compare simultaneously all known TGV prediction equations recommended for use with the Bod Pod. Second, our sample was similar in size to the original derivation studies of Crapo et al. (1982), Fields et al. (2004), and Ducharme et al. (2022), and was larger than most subsequent validity studies of these TGV prediction formulas. Further, our sample was diverse in height and body size leading to a wide range of lung volumes and %BF values. Nevertheless, there are several study limitations. First, measured TGV in the Bod Pod is an estimate based on plethysmography principles and relies on physiological assumptions (e.g., isothermal gas compression, airway compliance, and pressure transmission) (Criee et al. 2011; Wanger et al. 2005). Therefore, the observed agreement between DUCHARME and measured TGV may reflect convergence of estimation errors. We did not include an alternative comparison method, such as another body plethysmograph (e.g., Body Box) or gas dilution (e.g., helium dilution or nitrogen washout). However, previous research demonstrated strong reliability and validity of Bod Pod measured TGV compared to gas dilution techniques (Davis et al. 2007). Second, the findings from this study are limited to young adult athletes.

In summary, all of the TGV prediction equations have a bias such that individuals with a small TGV are overpredicted and those with a large TGV are underpredicted. Consequently, it is best to measure TGV when possible. A measured TGV is imperative if accuracy of a Bod Pod assessment is critical to a study or athlete evaluation. If using the Bod Pod to track longitudinal changes in body composition, a predicted TGV may be acceptable because the error will be constant from pre-test to post-test. If TGV must be predicted, the formula of Ducharme et al. (2022) (DUCHARME) is recommended for adults. Although a bias exists for DUCHARME, this formula blunts the magnitude of error compared to other prediction formulas, particularly for individuals at the extremes of the TGV continuum. Fewer individuals were misestimated by ≥ 2% BF when using DUCHARME compared to CRAPO or FIELDS in a sample of young adult athletes. Thus, we recommend that the Bod Pod manufacturer replace the CRAPO equation with the DUCHARME equation as the default prediction for TGV in their software.

## Data Availability

The dataset is publicly available on the figshare repository: 10.6084/m9.figshare.24094278.v1

## Abbreviations

ADP: Air displacement plethysmography
SEE: Standard error of estimate
TGV: Thoracic gas volume
%BF: Percentage of body fat
